# The Emerging Role of Intelectin-1 in Cancer

**DOI:** 10.3389/fonc.2022.767859

**Published:** 2022-02-03

**Authors:** D. Robert Paval, Thomas George Di Virgilio, Richard J. E. Skipworth, Iain J. Gallagher

**Affiliations:** ^1^ Faculty of Health Sciences and Sport, University of Stirling, Stirling, United Kingdom; ^2^ Clinical Surgery, University of Edinburgh, Royal Infirmary of Edinburgh, Edinburgh, United Kingdom

**Keywords:** omentin, adipokine, cancer, tumor, intelectin, gastrointestinal

## Abstract

**Background:**

Intelectin (ITLN) is an adipokine with two homologs—ITLN1 and ITLN2—that has various physiological functions. Studies analyzing the relationship between ITLN and cancer are focused on ITLN1; the available literature on ITLN2 and cancer is limited. This review aims to evaluate the role of ITLN1 in cancer without imposing any inclusion criteria, to examine pro- and anticancer roles for ITLN1 and to discuss whether the relationship between ITLN and cancer is mediated by obesity.

**Findings:**

Overall, ITLN1 level was highly variable in cancer patients but different from healthy individuals. Compared with control groups, patients with gastrointestinal and prostate cancer showed increased concentrations of circulating ITLN1, while patients with gynecological, breast, bladder, and renal cancer had lower ITLN1 levels. Several studies also evaluated tissue and tumor expression of ITLN1. In gastrointestinal cancer, ITLN1 was increased in tumor tissue compared with adjacent healthy tissue and elevated in the visceral adipose tissue of patients compared with controls. Consequently, the high levels of circulating ITLN1 might be determined by the tumor and by the cancer-associated weight loss in gastrointestinal cancer. ITLN1 can activate the phosphoinositide-3-kinase-protein kinase B/Akt (PI3K/Akt) pathway. The improper regulation of this pathway may contribute to a series of cellular events that favor tumor development and progression. Obesity has been linked with an increased risk of developing some cancers. Indeed, low circulating ITLN1 levels may be a marker of the metabolic effects of obesity, rather than obesity per se, and might contribute to a deregulation of the PI3K/Akt pathway.

**Conclusions:**

ITLN1 could be associated with cancer formation and progression. Since circulating ITLN1 levels are highly variable and differ between cancer types, the local tumor production of ITLN1 could be more relevant in determining malignant behavior. Future research should aim to identify the source of ITLN1 variability, to understand the differences in ITLN1 between distinct tumor types, and to further explore the signaling pathways through which this adipokine influences cancer biology.

## Introduction

Intelectin (ITLN), also known as omentin, is a 34-kDa lectin that contains a fibrinogen-like domain and a unique intelectin-specific region that makes it distinct from other immune lectins (1, 2). There are two homologs with 83% amino acid identity termed intelectin-1 (omentin-1) and intelectin-2 (omentin-2). ITLN is mainly produced by stromal vascular fraction cells, but not by adipocytes, in visceral adipose tissue, while its levels are very low in subcutaneous adipose tissue (3). Moreover, ITLN1 expression has also been found in epicardial fat, the small intestine, colon, ovary, lungs, and renal collecting tubes, whereas ITLN2 is expressed in intestinal Paneth cells (2, 4, 5). Both ITLN homologs can bind microbial glycan chains but not human glycans, and thus, the adipokine may have a role in antimicrobial defense (2, 6). Previous work has also identified the possible roles for ITLN1 in polyspermy prevention and in iron metabolism *via* interaction with lactoferrin (2).

The interest in the role of ITLN in cancer has been driven by the observation that ITLN1 levels differ between healthy individuals and patients with various types of cancer (7–10). We recently identified ITLN1 and ITLN2 mRNA as increased in the visceral adipose tissue of gastrointestinal cancer patients and found that local but not circulating ITLN1 protein demonstrated a relationship with cancer cachexia (11). It has also been suggested that the relationship between ITLN1 and cancer might be influenced by overweight and obesity (12). Collectively, these findings indicate that ITLN might have a role in cancer biology and that it could be used as a biomarker for cancer itself or cancer progression. Yet, the exact mechanisms through which this adipokine induces physiological changes are not completely understood, and the variable levels of ITLN described in the literature have not been fully explained by previous research. Therefore, we will review the available literature on ITLN in cancer without imposing any inclusion criteria, discuss the proposed pro- and anticancer roles for ITLN, and finally, analyze whether the relationship between ITLN and cancer is mediated by body mass index (BMI). Furthermore, this review will identify current knowledge gaps and propose pathways for future work. Most of the studies analyzing the relationship between ITLN and cancer focus on ITLN1. Although ITLN2 is likely to influence various physiological processes, there are little published data on this topic, and therefore, the present review will focus on ITLN1.

## Discussion

### ITLN1 and Gastrointestinal Cancers

A recent systematic review examining circulating ITLN1 levels in cancer found that ITLN1 was often increased in individuals with colorectal cancer compared with healthy controls (12). Another study evaluating a cohort of Chinese patients identified increased ITLN1 levels as a potential risk factor for colorectal cancer (13). Higher levels of circulating ITLN1 were also found to increase the probability of recurrence or death in colorectal cancer after surgery; yet, the difference in circulating ITLN1 between the groups was very small (14). A study by Ummugul Uyeturk and colleagues (15) examined circulating ITLN1 levels after surgery and chemotherapy in colorectal cancer and found that the levels remained elevated compared with a healthy control group who did not have surgery or chemotherapy. However, the reported range of results in this study was very low (pg/ml). Circulating ITLN1 levels also seem to be increased in pancreatic cancer patients compared with healthy controls (8, 16). In comparison, two studies have found that higher tumor expression (rather than circulating levels) of ITLN1 was associated with a good prognosis in colorectal cancer (17, 18). Unfortunately, circulating ITLN1 was not assessed in these studies. Zheng and colleagues (19) emphasized that ITLN1 levels were greater in gastric cancer tissue compared with normal gastric mucosa. We previously identified higher ITLN1 expression in the visceral adipose tissue of patients with upper gastrointestinal cancer compared with healthy controls, while the expression of ITLN1 mRNA in subcutaneous adipose tissue and circulating ITLN1 levels did not differ between groups (11).

Although the evidence is not strong enough to generate a definitive conclusion, it can be argued that ITLN1 expression is increased locally and perhaps systemically in gastrointestinal cancers. The elevated circulating levels of this adipokine might be partly determined by the tumor (20) and the degree of cancer-associated weight loss (11, 21). While the function of ITLN1 in these cancers is unknown, some of the evidence presented above would suggest that higher local tumor levels may be a good prognostic indicator. However, the reported circulating levels of ITLN1 are extremely variable. Thus, while ITLN1 may have potential as a biomarker for the diagnosis or progression of gastrointestinal cancers, circulating levels may be too variable to be useful. This question should be investigated with appropriately powered studies.

### ITLN1 and Urological Cancers

The levels of ITLN1 are also dysregulated in prostate cancer. Arjmand and colleagues (12) suggested that prostate cancer patients had significantly higher circulating ITLN1 levels compared with control groups in all the studies included in their review. Although the stated aim of their review was to compare cancer patients with healthy controls, not all of the included studies respected this criterion. In the prostate cancer subgroup analysis, two studies compared cancer patients against individuals with benign prostatic hyperplasia (BPH). In both studies, circulating ITLN1 was higher in prostate cancer than in BPH (22, 23). Similarly, a more recent study observed that prostate cancer patients had greater concentrations of circulating ITLN1 compared with individuals with BPH (24). Zhang et al. (25) examined circulating ITLN1 levels in bladder cancer and found reduced levels in patients compared with healthy controls. However, the reported ITLN1 levels were outside the assay range indicated in the methodology of the study. Circulating ITLN1 was also lower in patients with renal cell carcinoma than in matched controls (26). In summary, the available literature suggests that circulating ITLN1 is elevated in prostate cancer and reduced in bladder and renal cancer. Further evidence is required to strengthen these interim conclusions.

### ITLN1 and Breast and Gynecological Cancers

Three reports examined the circulating levels of ITLN1 in breast cancer patients compared with healthy controls. Patients with breast cancer had lower circulating ITLN1 levels in all three studies (10, 27, 28). Tahmasebpour and colleagues (10) also observed that ITLN1 gene expression was significantly downregulated in breast cancer tissue compared with adjacent normal tissue. Several studies assessed the relationship between ITLN1 and gynecological cancers. In a subgroup meta-analysis by Arjmand et al. (12), circulating ITLN1 did not significantly differ between women with ovarian cancer and healthy controls. Another study evaluating the same topic (29) indicated that circulating ITLN1 was lower in patients with high-grade ovarian cancer as opposed to healthy women and women with benign gynecological disease. Moreover, these authors observed that ITLN1 mRNA was expressed at a lower level in the omental adipose tissue of patients with high-grade ovarian cancer compared with women with benign disease (29). Interestingly, this finding contradicts our study in upper gastrointestinal cancer (11) where disease severity increased omental ITLN1 expression. Holman et al. (30) and Cymbaluk-Ploska et al. (31) also observed lower circulating ITLN1 in women with endometrial cancer. Although both studies were analyzed in the systematic review by Arjmand and colleagues (12), only Holman et al. (30) compared cancer patients with healthy controls. The comparison group from the other study (31) included women with endometrial polyps (benign endometrial changes). In summary, despite the fact that only a limited amount of data were available, a tendency for lower circulating ITLN1 levels in breast and gynecological cancers was observed. Future studies should aim to explore this relationship and to develop a better understanding of the role of ITLN1 in these types of cancer.

### Other Cancers

In an examination of 42 neuroblastoma tumors, Li et al. (32) found that ITLN1 protein was expressed in 33% of the tumor specimens with variable degrees of staining, from weak to intense. Higher levels of ITLN1 staining tended to occur in tumors with more favorable features (32). Furthermore, data mining of the R2 microarray genomics and visualization resource suggested that higher ITLN1 mRNA expression was associated with a greater probability of survival (32). Another study noted a lower level of circulating ITLN1 in Iranian male smokers with lung cancer compared with apparently healthy smokers. Notably, circulating ITLN1 in smokers with lung cancer was not different from that of healthy non-smokers (33). An *in-silico* meta-analysis of RNA-Seq data noted that ITLN1 mRNA expression was consistently lower in lung tumors compared with healthy lung tissue across seven data sets and that higher ITLN1 mRNA levels in tumors predicted better survival (34). In malignant pleural mesothelioma (MPM), the early work by Wali et al. (35) found that ITLN1 mRNA and protein were highly expressed in tumors as opposed to normal tissue. Subsequently, increased expression of ITLN1 was observed in mesothelioma cell lines and epithelioid mesotheliomas (36–38). ITLN1 levels were elevated in the pleural fluid of MPM patients compared with the levels seen in lung cancer, tuberculosis, or pneumonia (36). The mean differences were driven by a few individuals with very high levels of pleural ITLN1 (>3,000 ng/ml), while plasma ITLN1 concentrations did not differ between MPM patients and healthy controls (36). Although immunoreactivity for ITLN1 in epithelioid mesothelioma tumors was very strong, ITLN1 seemed to be absent from other tumors (both mesothelioma and non-mesothelioma) unless they were mucus-producing (36–38). Overall, the available data on the previously discussed types of cancer are limited but generate pathways for further research. The direction of the difference in circulating ITLN1 between cancer patients and control groups depends on cancer type. Similarly, different tumors were found to express distinct concentrations of ITLN1. **Table S1** in the **Supplementary Materials** section includes a summary of the studies from this review that measured ITLN1 in human participants diagnosed with cancer. The source of ITLN1 variability between different types of cancer should be one of the main areas explored by future studies.

### Mechanisms for Intelectin Effects in Cancer

**Figure 1:**
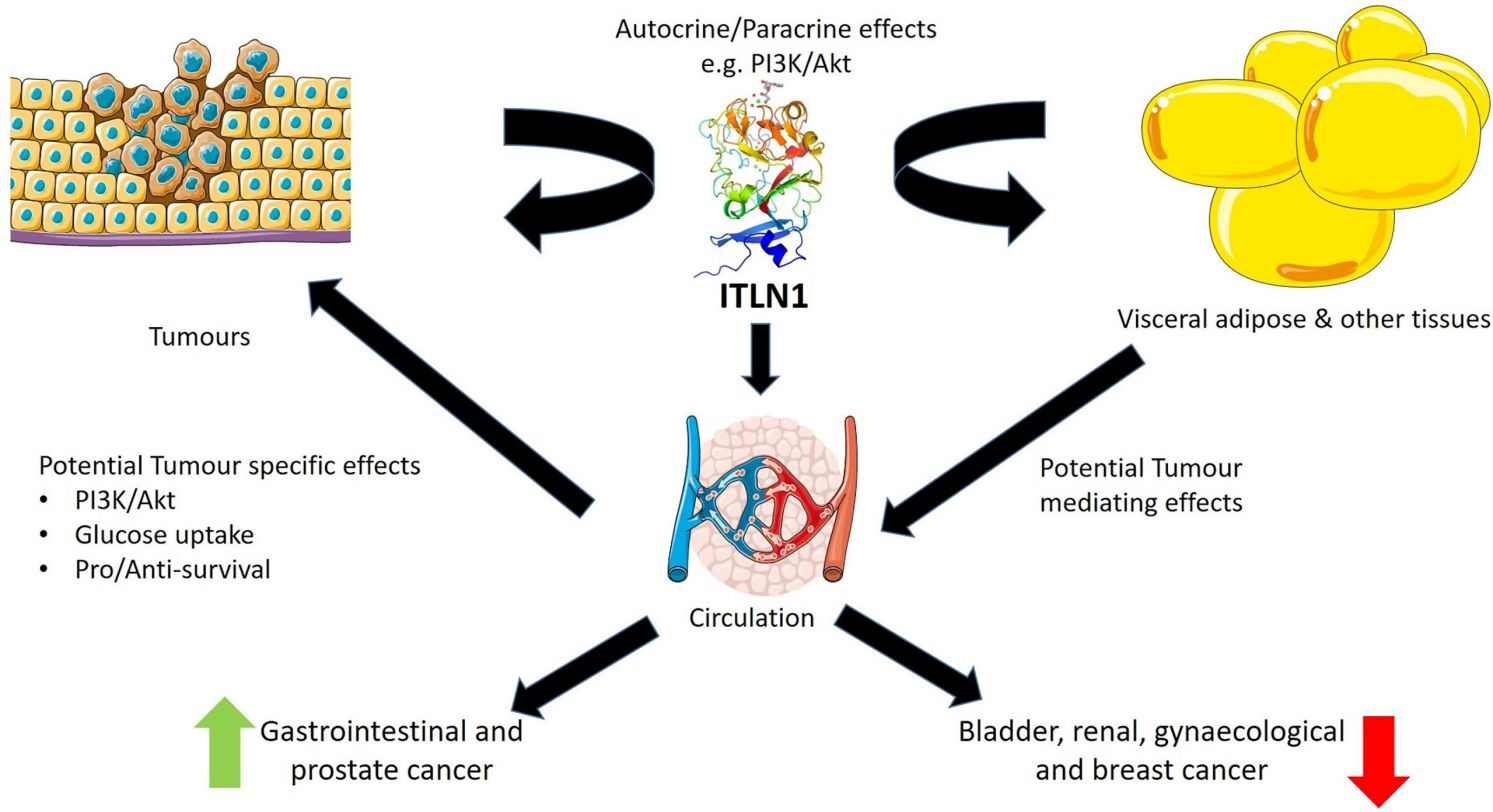
Potential roles for intelectin-1 (ITLN1) in cancer. ITLN1 produced by tumors may have pro- or antisurvival autocrine and/or paracrine effects on these tumors. Simultaneously, ITLN1 produced by normal tissues such as the visceral adipose tissue or the premalignant/malignant tissue may enter the circulation and affect tumor biology *via* PI3/Akt or other pathways. Circulating ITLN1 is reduced with increased adiposity, and therefore, adiposity may act as a mediator of the effects of ITLN1 on tumors or tumor formation. Results in the literature suggest that, as a biomarker, circulating ITLN1 is increased in gastrointestinal and prostate cancer and decreased in bladder, renal, gynecological, and breast cancer. The clinical relevance of these findings requires further investigation. Image credits: ITLN1 structure—Kwangkanont on Wikimedia, CC-BY-SA; other images provided by Servier Medical Art under CC.BY 3.0. https://smart.servier.com.

**Figure 1** highlights the potential roles of ITLN1 in cancer and its probable mechanisms of action. Previous work has indicated that ITLN1 can influence Akt-mediated growth pathways. The phosphoinositide-3-kinase-protein kinase B/Akt (PI3K/Akt) signal transduction pathway has a crucial role in various cellular functions that contribute to cancer, including metabolism, growth, motility, proliferation, and angiogenesis (39). ITLN1 increases Akt phosphorylation in adipocytes, osteoblasts, and mesenchymal cells (3, 40, 41). Wu and colleagues (40) also indicated that the inhibition of Akt prevents the proliferative effect of ITLN1 in osteoblasts, suggesting that ITLN1 signals through the Akt pathway in these cells. In mesenchymal stem cells, ITLN1 also leads to Akt-mediated proliferation, resistance to oxidative stress, and secretion of proangiogenic factors (41). These actions may be particularly important in tumors as mesenchymal stem cells are part of the tumor microenvironment and can favor tumor growth (42). Activation of mesenchymal gene programs is also a characteristic of advanced tumors (43). Overall, these findings suggest that ITLN1 could locally potentiate PI3K/Akt signaling and glucose uptake in cancer cells to promote survival. The ITLN1/TMEM207 axis is another pathway that may influence carcinogenesis (44). TMEM207 is a transmembrane protein that has a role in ITLN1 processing. It was suggested that low levels of TMEM207 contribute to a decrease in ITLN1 concentrations and, consequently, promote colorectal carcinogenesis (44, 45). The available research on this pathway is still limited and future studies are needed to confirm the relationship between TMEM207 and ITLN1. Given that circulating ITLN1 varies between different types of cancer, the local level or the tumor production of ITLN1 may be more important than the circulating level in contributing to malignant behavior.

Conversely, other groups have found that ITLN1 may play an antitumor role. In colorectal cancer cell lines, the reduction of ITLN1 expression by short-hairpin RNA increased cell growth and proliferation as well as Akt, extracellular signal-regulated kinase (ERK), and epidermal growth factor receptor (EGFR) phosphorylation (18). ITLN1 treatment reduced the proliferation and the migratory ability of gastric cancer cells by upregulating hepatocyte nuclear factor 4α (HNF4a) *via* increased inhibition of nuclear factor kappa B (NFkB) (46). Furthermore, a supraphysiological dose of ITLN1 (1–2 μg/ml) led to the arrest of cell cycling and subsequent apoptosis in hepatocellular carcinoma cell lines (47). Supraphysiological ITLN1 also inhibited the proliferation and promoted the apoptosis of colon cancer stem cells in a time-dependent manner (48). In neuroblastoma cell lines and human tumors, increased ITLN1 seemed to have a PI3K/Akt-mediated protective effect by increasing the expression of N-myc downstream-regulated gene 2 (NDRG2) and by reducing tumor volume and metastatic potential (32). Notably, higher ITLN1 expression in human neuroblastoma tumors improved the probability of survival (32). Similarly, higher tumor expression of ITLN1 was associated with improved prognosis in patients with colorectal cancer (17, 18). These results suggest that ITLN1 may have potential as an anticancer agent. However, the levels of ITLN1 used in many of these studies were in the μg/ml range, whereas the normal physiological concentration of ITLN1, even in cancer or cell culture supernatant, was in the ng/ml range. Therefore, there may be unanticipated physiological effects if elevated levels of ITLN1 are used as an anticancer agent.

### Intelectin, Obesity, and Cancer Risk

Visceral adipose tissue is the primary source of ITLN suggesting that the relationship between this adipokine and various cancer mechanisms could be mediated by BMI or adiposity. The presence of excess adipose tissue alters adipokine production which may contribute to the development of cancer, indicating the potential role of ITLN1 (44, 49). Also, it has been previously demonstrated that obese individuals present a higher risk of developing cancer compared with people with normal weight (50). Obesity has been linked to lower levels of circulating ITLN1 in several studies (5, 51). However, low circulating ITLN1 levels in people with impaired glucose regulation and untreated type 2 diabetes (52), women with metabolic syndrome secondary to polycystic ovary syndrome (53), and Japanese men with a higher number of metabolic risk factors (54) as well as in women with gestational diabetes and BMI <30 (52) suggest that low ITLN1 may be a marker for the metabolic effects of obesity rather than obesity itself. Although a recent meta-analysis of studies examining the relationship between body weight and circulating ITLN1 levels found an overall effect, there was very high study heterogeneity (93%) and evidence of publication bias (55). Furthermore, subgroup analysis revealed that higher-quality studies suggested no relationship and that serum ITLN1 level was significantly lower in overweight but not obese individuals (55). In summary, the available evidence suggests that circulating ITLN1 may be a marker of metabolic dysregulation rather than overweight/obesity per se.

In their review, Arjmand and colleagues (12) found no overall relationship between circulating ITLN1 and cancer. As we note above, evidence suggests that the direction of change for circulating ITLN1 may be different in the presence of different tumors. Notably, levels of circulating ITLN1 were consistently higher than control groups in gastrointestinal and prostate cancer and lower than control groups in renal, lung, bladder, endometrial, and breast cancer (12). Arjmand et al. (12) also suggested that ITLN1 levels were higher in studies examining cancer patients with a mean BMI >25 as opposed to studies that included individuals with an average BMI <25. This could indicate an interaction between cancer presence, adiposity, and intelectin. However, as noted above, the evidence base for an association between circulating ITLN1 and overweight/obesity is weak (55). Arjmand et al. (12) also grouped studies based on average BMI values but did not consider the variation around the mean for several studies (7, 9, 22, 28, 33, 56). Not all of the patients included in these studies had a BMI >25 according to their published variance statistics. Including these studies in the “BMI >25” subgroup therefore does not represent a valid statistical approach. Thus, tumor-specific directional changes in circulating ITLN1 levels, local versus global changes in ITLN1, and aggregate views of overweight/obesity or metabolic status in patient groups could lead to misinterpretations of the relationship between circulating ITLN1 and cancer risk or status.

### Future Directions

Changes in circulating ITLN1 levels have been associated with several cancers. Tumor site and/or type seems to be important in the direction of the change. Further work is required to identify whether ITLN1 has utility as a biomarker for cancer occurrence or re-occurrence. This work would have to further confirm and take account of the possible tumor-specific direction of change in ITLN1. As we noted in our work on adipose tissue in upper gastrointestinal cancer, increased production of visceral adipose tissue ITLN1 is a feature of cancer-induced weight loss (11). Future studies with larger cohort sizes should also investigate whether cancer-induced weight loss leads to increased circulating ITLN1. The mechanisms by which ITLN1 may lead to either positive or negative change in cancer also deserve research attention. Further work on the signaling pathways and the roles of ITNL1 in both normal and dysregulated metabolism should help identify the mechanisms through which this adipokine influences tumor biology and the metabolic consequences of cancer.

## Conclusion

The available literature indicates that ITLN1 might have a role in cancer formation and development since ITLN1 level was highly variable but different from healthy controls when patients with various cancer types were examined. High concentrations of ITLN1 were found in patients with gastrointestinal (8, 13) and prostate cancer (23) compared with control groups. Conversely, women with breast (27) and gynecological cancer (29) expressed less ITLN1 as opposed to healthy individuals. Although these are relevant observations, a meta-analysis with a reliable methodological approach is required to quantify ITLN1 levels in cancer patients and healthy participants. Most available studies, including a recent systematic review (12), only measured differences between groups. To date, no study discussed the variability in ITLN1 levels observed in both healthy and diseased individuals. For instance, in the review by Arjmand et al. (12), mean ITLN1 varied from 1.8 to 618.0 ng/ml in the cancer groups, while the range in the control groups was 1.6 to 756.4 ng/ml. Therefore, the source of ITLN1 variability and its physiological concentration should be determined by future research.

Several studies (40, 41) assessed the mode of action of ITLN1 and observed that it activates the PI3k/Akt pathway. The improper regulation of this pathway could lead to the proliferation of mesenchymal cells that favor the progression and development of cancer. Low ITLN1 levels are a marker of the metabolic effects of obesity and might contribute to a deregulation of the PI3k/Akt pathway. Otherwise, local changes in ITLN1 could favor a protumor environment. Further research is required to clarify the role of ITLN1 in carcinogenesis and to describe the pathways through which this adipokine interacts with other cells. Moreover, future studies should aim to assess ITLN1 expression in cancer patients by measuring its concentration in the blood, cancer tissue, and subcutaneous and visceral adipose tissue. Animal models and *in-vitro* investigations might focus on determining the direction of the causal relationship between ITLN1 and cancer. This area of research has been emerging during recent years and there are numerous promising pathways that can be explored.

## Author Contributions

DP: conceptualization, methodology, writing of the drafts and the final version of the manuscript, and editing. TV: conceptualization and writing of the drafts and the final version of the manuscript. RS: conceptualization and writing of the drafts and the final version of the manuscript. IG: conceptualization, methodology, writing of the drafts and the final version of the manuscript, editing, and supervision. All authors contributed to the article and approved the submitted version.

## Conflict of Interest

The authors declare that the research was conducted in the absence of any commercial or financial relationships that could be construed as a potential conflict of interest.

## Publisher’s Note

All claims expressed in this article are solely those of the authors and do not necessarily represent those of their affiliated organizations, or those of the publisher, the editors and the reviewers. Any product that may be evaluated in this article, or claim that may be made by its manufacturer, is not guaranteed or endorsed by the publisher.
