# Supplementary material for: The Emerging Role of Intelectin-1 in Cancer
Source: Front Oncol. 2022 Feb 3;12:767859. doi: 10.3389/fonc.2022.767859 (PMC8850632; doi:10.3389/fonc.2022.767859)
Supplement: Supplementary file 1 [file Table_1.docx]

**Supplementary material**

**Table 1. Summary table - characteristics of studies that examined human participants with different types of cancer**

| **Study (year)** | **Cancer type** | **Participants** | **Findings** | **ITLN1 source** | **ITLN1 level in cancer** |
| --- | --- | --- | --- | --- | --- |
| **Gastrointestinal cancers** | | | | | |
| Zhao et al. (2019) [13] | Colorectal | 358 patients  286 controls | Increased ITLN1 associated with a higher risk of developing colorectal cancer. | Circulating | ↑ |
| Feng et al. (2020) [14] | Colorectal | 319 patients  300 controls | Higher levels of ITLN1 increase the probability of recurrence or death after surgery. The difference between patients and controls was small. | Circulating | ↑ |
| Um. Uyeturk et al. (2014) [15] | Colorectal | 45 patients  35 controls | Increased ITLN1 levels after surgery and chemotherapy compared to a healthy control group. | Circulating | ↑ |
| Karabulut et al. (2016) [8] | Pancreatic | 33 patients  30 controls | ITLN1 levels increased in patients compared to healthy controls. | Circulating | ↑ |
| Kiczmer et al. (2018) | Pancreatic | 20 patients  18 controls | ITLN1 elevated in patients compared to healthy controls. | Circulating | ↑ |
| Miller et al. (2020) [11] | Upper gastrointestinal | 16 patients  8 controls | ITLN1 levels did not differ between groups. | Circulating | ↔ |
|  |  |  | Higher ITLN1 expression in the visceral adipose tissue of patients compared to healthy controls. No difference in subcutaneous adipose tissue expression. | Tissue | ↑ |
| Kim et al. (2012) [17] | Colorectal | 6 patients | Higher tumour expression of ITLN1 was associated with good prognosis. | Tissue | ↓ |
| Katsuya et al. (2020) [18] | Colorectal | 148 patients | Increased tumour expression of ITLN1 was associated with good prognosis. | Tissue | ↓ |
| Zheng et al. (2012) [19] | Gastric | 196 patients | ITLN1 level was greater in gastric cancer tissue compared to normal gastric mucosa. | Tissue | ↑ |
| **Urological cancers** | | | | | |
| Ug. Uyeturk et al. (2014) [22] | Prostate | 50 patients  30 controls | ITLN1 was higher in patients than in controls suffering from benign prostate hyperplasia. | Circulating | ↑ |
| Fryczkowski et al. (2018) [23] | Prostate | 40 patients  40 controls | ITLN1 was higher in patients compared to controls with benign prostate hyperplasia. | Circulating | ↑ |
| Borowski and Sieminska (2020) [24] | Prostate | 72 patients  65 controls | Patients had greater ITLN1 concentrations compared to individuals with benign prostate hyperplasia. | Circulating | ↑ |
| Zhang et al. (2016) [25] | Bladder | 42 patients  42 controls | ITLN1 levels were decreased in patients compared to healthy controls. | Circulating | ↓ |
| Shen et al. (2016) [26] | Renal cell carcinoma | 41 patients  42 controls | ITLN1 levels were lower in patients as opposed to healthy controls. | Circulating | ↓ |
| **Breast and gynaecological cancers** | | | | | |
| Alaee et al. (2016) [27] | Breast | 30 patients  30 controls | Patients with breast cancer had lower ITLN1 levels compared to healthy controls. | Circulating | ↓ |
| Nourbakhsh et al. (2018) [28] | Breast | 45 patients  45 controls | Patients with breast cancer had lower ITLN1 levels compared to healthy controls. | Circulating | ↓ |
| Holman et al. (2014) [30] | Endometrial | 74 patients  74 controls | Lower ITLN1 levels in patients compared to healthy controls. | Circulating | ↓ |
| Cymbaluk-Ploska et al. (2018) [31] | Endometrial | 92 patients  76 controls | Decreased ITLN1 in patients compared to controls. The control group included 32 women with endometrial polyps and 44 with normal endometrium. | Circulating | ↓ |
| Tahmasebpour et al. (2002) [10] | Breast | 88 patients  86 controls | Patients had lower ITLN1 levels compared to healthy controls. | Circulating | ↓ |
|  |  |  | ITLN1 gene expression was significantly downregulated in breast cancer tissue compared to adjacent normal tissue | Tissue | ↓ |
| Au-Yeung et al. (2020) [29] | Ovarian | 147 patients  147 controls | ITLN1 was lower in patients as opposed to healthy women (n=99) and women with benign gynaecological disease (n=48). | Circulating | ↓ |
|  |  |  | ITLN1 mRNA was expressed at a lower level in the omental adipose tissue of patients compared to women with benign disease. | Tissue | ↓ |
| **Other cancers** | | | | | |
| Ansari et al. (2018) [33] | Lung | 45 patients  61 controls | ITLN1 levels did not differ between patients and healthy non-smokers. | Circulating | ↔ |
| Tsuji et al. (2010) [36] | Malignant pleural mesothelioma | 8 patients | ITLN1 concentrations did not differ between patients and healthy controls. | Circulating | ↔ |
| Li et al.  (2015) [32] | Neuroblastoma | 42 patients | Higher levels of ITLN1 observed in tumours with more favourable characteristics. Higher ITLN1 mRNA expression associated with a greater probability of survival. | Tissue | ↓ |

The arrows are used to indicate the level of ITLN1 in patients (or cases with a worse prognostic/ higher risk of cancer) compared to controls (or cases with improved prognostic/ lower risk of cancer).
